# Altered hippocampal lipid profile underlies anxiety-like behavior secondary to mild traumatic brain injury

**DOI:** 10.1007/s11010-026-05677-w

**Published:** 2026-08-08

**Authors:** Celyne Moreira Olimpo, Caroline Amaral Machado, Isadora Helena Pimenta Veloso, Pedro Nepomuceno Parodi, Ana Carolina Feijó Cerqueira Kiryu, Ricardo Tadeu de Carvalho, Antônio L. Teixeira, Aline Silva de Miranda, George E. G. Kluck

**Affiliations:** 1 Department of Morphology, Institute of Biological Science, Federal University of Minas Gerais, Belo Horizonte, MG Brazil; 2 Center for Discovery and Innovation - The City University of New York, New York, NY USA; 3 McGovern Medical School, University of Texas Health Science Center at Houston, Houston, TX USA

**Keywords:** Trauma brain injury, Anxiety, Lipids, Metabolism

## Abstract

Traumatic brain injury (TBI) remains a major cause of neurological morbidity worldwide, and mild TBI (mTBI) is frequently associated with emotional disturbances, including anxiety-like behavior. Although classical mechanisms of secondary injury have been extensively characterized, metabolic alterations, particularly those involving cerebral lipid homeostasis, remain incompletely defined. Given the structural and regulatory roles of lipids in the central nervous system, disruption of lipid metabolism may contribute to early post-traumatic dysfunction. We hypothesized that mTBI induces region-specific alterations in brain lipid metabolism and that these changes are associated with anxiety-like behavior during the acute post-injury phase. Adult male C57BL/6 mice were subjected to a weight-drop model of mTBI and evaluated 48–72 h post-injury using different behavioral paradigms. Lipid classes were quantified in the hippocampus, prefrontal cortex, and serum by thin-layer chromatography. mTBI induced anxiety-like behavior without locomotor impairment. Biochemical analyses revealed region-dependent lipid alteration predominantly in the ipsilateral hippocampus, characterized by reduced diacylglycerol and monoacylglycerol levels alongside increased total phospholipids and specific subclasses. Correlation analyses demonstrated significant associations between anxiety-related behavioral parameters and lipid classes across tissues. These findings indicate that mild TBI is accompanied by early, region-specific lipid alterations that are associated with behavioral changes during the acute post-injury period.

**Supplementary Information:**

The online version contains supplementary material available at https://doi.org/10.1007/s11010-026-05677-w.

## Introduction

Traumatic brain injury (TBI) is defined as a brain injury resulting from the application of an external force to the skull, leading to structural and functional alterations in the central nervous system (CNS) [1]. It represents a major public health concern worldwide, being strongly associated with high morbidity, long-term disability, and the need for prolonged medical care [2]. In the United States alone, approximately 3 million emergency department visits per year are attributed to TBI, with nearly 120,000 cases progressing to permanent disabilities [2, 3].

Mild TBI (mTBI) accounts for approximately 80–90% of all TBI cases, and although often classified as less severe, it is frequently associated with persistent symptoms [4, 5]. Long-term consequences of mTBI include cognitive deficits, behavioral and emotional symptoms, which significantly affect functional capacity and quality of life [6–8]. The development of TBI-related complications is closely related to the progression of secondary brain injury mechanisms, in which the primary insult triggers a cascade of cellular and molecular events, including excitotoxicity, oxidative stress, mitochondrial dysfunction, and neuroinflammation [9–12]. While these mechanisms are recognized contributors to secondary injury, increasing evidence suggests that metabolic disturbances, including alterations in lipid homeostasis, may also contribute to secondary injury mechanisms following mTBI [13–15].

The CNS is one of the most lipid-enriched tissues in the body, with lipids accounting for nearly half of its dry weight. In addition to their structural roles in myelin formation and membrane organization, lipids are essential regulators of synaptic transmission, neuronal excitability, neuroinflammation, and cellular responses to injury [16–18]. Following TBI, activation of phospholipases, oxidative stress, and mitochondrial dysfunction can profoundly disrupt lipid homeostasis, triggering widespread alterations in phospholipid, sphingolipid, glycerolipid, and fatty acid metabolism. Recent evidence indicates that mTBI is associated with degradation and remodeling of membrane phospholipids, such as phosphatidylcholines, as well as increased release of free fatty acids [19–21]. These alterations may affect membrane integrity, impair synaptic plasticity, and disrupt metabolic homeostasis, thereby contributing to neurological dysfunction after trauma. Importantly, such lipid disturbances have been reported in both moderate and mild injury models and are detectable from the acute post-injury period through chronic stages [14, 15, 22, 23]. Although lipid alterations following mTBI have been described, most previous studies have focused on characterizing biochemical changes or identifying potential biomarkers of injury [14, 24–26]. It remains unclear whether post-traumatic lipid disturbances are associated with the behavioral abnormalities observed after mTBI. Based on these observations, we hypothesized that mTBI would induce region-specific changes in brain lipid composition during the acute post-injury phase and that these changes would be associated with anxiety-like behavior. To test this hypothesis, we used an established weight-drop mTBI model in mice to characterize lipid profiles across distinct brain regions and evaluate their relationships with behavioral outcomes. Understanding the association between alterations in cerebral lipid metabolism and emotional manifestations, such as anxiety- and depression-like behaviors, may provide further insight into the biological processes underlying TBI-related sequelae.

## Materials and methods

### Animals

**Fig. 1:**
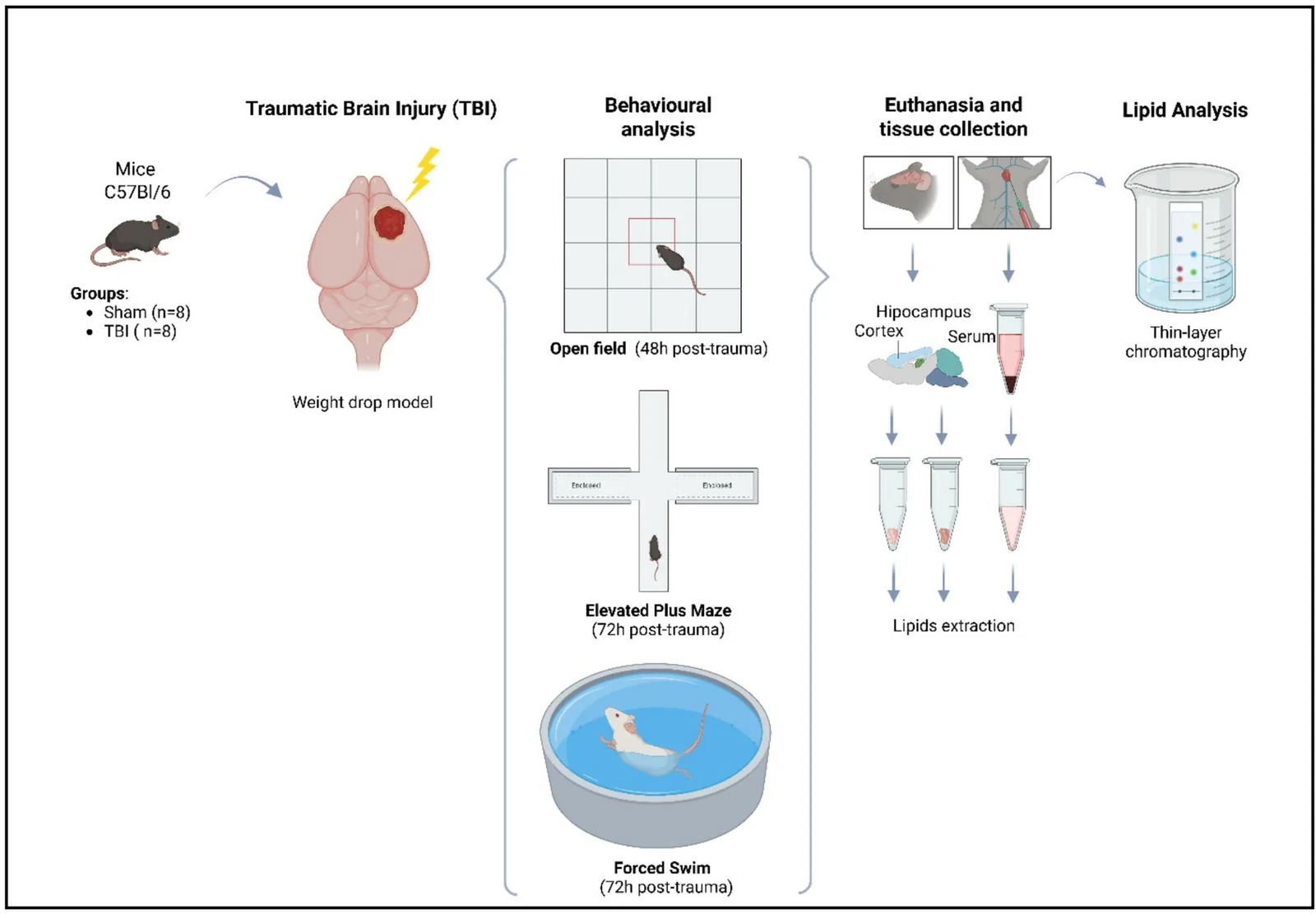
Schematic representation of the experimental design. Created in BioRender. Imperio, G. (2026) https://BioRender.com/yuokp28

Adult male C57BL/6 wild-type mice (20–30 g), aged 10–12 weeks, were supplied by the Animal Facility Center of the Federal University of Minas Gerais (UFMG), Belo Horizonte, Brazil, and randomly divided into two groups: (1) Sham (*n* = 8) and (2) Traumatic brain injury (TBI) (*n* = 8). All experimental procedures were approved by the Ethics Committee on Animal Experimentation of UFMG (protocol number 275/2024). Animals were housed under controlled conditions with a 12 h light/dark cycle and had free access to food and water. Before the behavioral and cognitive assessments, mice were allowed to acclimate to the testing environment for at least 30 min. The overall experimental timeline is illustrated in Fig. 1.

### Mild TBI injury

The weight-drop injury protocol applied in this study was based on a model routinely performed at our laboratory [27]. Briefly, mice (TBI group) were anesthetized with a ketamine (80 mg/kg)/xylazine (10 mg/kg) mixture and positioned in the prone orientation. Following anesthesia, a midline scalp incision was performed to expose the skull, and a 15 g weight/3 mm tip was released through a 60 cm vertical guide tube, impacting the right parietal region between the sagittal and coronal sutures [28, 29]. Importantly, the mouse has the head fixed to ensure that the weight falls exactly at the same region of the skull. Each animal was subjected to a single mTBI or assigned to the sham condition. After the injury, the incision was closed, and the animals were continuously monitored until full recovery from anesthesia. During this period, mice were maintained on a heated surface and received ocular lubrication to prevent corneal dehydration. No postoperative fluid replacement was required, as all animals recovered uneventfully and showed no signs of dehydration or clinically relevant body weight loss. Classification of the trauma as mild was based on the combination of impact parameters (weight and drop height) and the absence of mortality, skull fractures, or significant body weight loss. As previously characterized [27], animals exhibited only transient neurological deficits, as assessed by the Neurological Severity Score (NSS), with recovery occurring within the first hours after injury, further supporting the classification of the model as a mTBI. Sham animals underwent all experimental procedures except for the weight-drop impact.

### Behavioral analyses

#### Open field test

Forty-eight hours following mTBI, locomotor activity was evaluated using the Open Field test implemented through the PhenoTyper system (Noldus Information Technology, Leesburg, VA, USA) [30]. Animals were placed individually in an open arena measuring 30 cm × 30 cm × 35 cm, and their spontaneous activity was recorded for 30 min. The total distance traveled during the test was quantified using EthoVision XT tracking software (Noldus Information Technology, Leesburg, VA, USA). The percentage of time spent at the open field center was also calculated as an indirect measure of anxiety-like behavior.

#### Elevated plus maze test

Anxiety-like behavior was evaluated using the elevated plus maze test (72 h after mTBI). Each animal was tested for a 5-minute session, and behavioral data were recorded and analyzed with EthoVision XT software (Noldus Information Technology, Leesburg, VA, USA). Decreased proportion of entrance and time spent at the open arms of the maze indicates anxiety-like behaviors [31]. The absolute number of entries in the closed arms of the maze was used as a measure of locomotor activity [31].

#### Forced swimming test

Depressive-like behavior was assessed using the forced swimming test (72 h after mTBI), following previously established protocols [30]. Briefly, mice were individually placed in a cylindrical glass container (18 cm diameter, 28 cm height) filled with water to a depth of 18 cm, and their behavior was recorded over a 6 min session. Behavioral scoring was restricted to the final 4 min of the test. Within this interval, immobility duration and latency to the first immobility episode were analyzed with EthoVision XT software (Noldus Information Technology, Leesburg, VA, USA) and quantified as indices of depressive-like behavior.

The same cohort of animals underwent all behavioral assessments. Behavioral testing was performed in a fixed sequence, with the open field test conducted first, followed by the elevated plus maze 24 h later and the forced swim test after a 2-h interval. All behavioral analyses were performed by a single experimenter blinded to the experimental groups.

### Tissue and blood collection

After behavioral assessments (72 h after mTBI), mice were anesthetized with an intraperitoneal injection of ketamine (80 mg/kg)/xylazine (10 mg/kg). Blood samples were collected by cardiac puncture using a 1-mL insulin syringe (100 IU) fitted with a fixed 30G needle (0.30 × 8 mm; Cepalab, Brazil). Following blood sampling, animals were euthanized by cervical dislocation. The skull was opened, and brain tissues, including the prefrontal cortex and hippocampus, were carefully dissected. Brain samples were immediately stored at − 80 °C. Blood samples were centrifuged in a refrigerated centrifuge at 400 x g for 10 min. Serum was collected into new microcentrifuge tubes and stored at − 80 °C.

### Lipid extraction and quantification

Lipid extraction was performed using a modified Bligh and Dyer method [32]. Samples (2 mg) of the prefrontal cortex and hippocampus were homogenized in a lysis buffer (50 mM Tris-HCl, pH 7.4, containing 1% NP-40, 250 mM NaCl, 5 mM EDTA, and 50 mM NaF) and then subjected to lipid extraction. For this, samples were transferred to conical glass tubes containing a chloroform-methanol-water solution in a 2:1:0.8 (v/v/v) ratio. The tubes were subjected to intermittent shaking for 2 h (5 min each interval), followed by centrifugation at 230 × g for 20 min at 4 °C in a refrigerated centrifuge (Hitachi, Ltd., Tokyo, Japan). The supernatant was collected, and 1 mL of water and 1 mL of chloroform were added to it. After thorough mixing, the samples were separated into two distinct phases (aqueous and organic). Water-soluble components of the homogenization buffer, including salts, Tris, EDTA, and detergent residues, remained in the aqueous phase, whereas lipids partitioned into the organic phase. The tubes were centrifuged again under the same conditions (230 × g, 20 min, 4 °C). The organic phase, containing the extracted lipids, was carefully removed and dried under a stream of nitrogen gas. The extracted lipids were analyzed by thin-layer chromatography (TLC) using a hexane-diethyl ether-acetic acid (60:40:1, v/v/v) mobile phase. Commercial lipid standards were applied to each TLC plate at three known concentrations and developed alongside tissue samples. These standards were used to identify lipid classes by their migration patterns and to generate calibration curves for quantification. Band intensity was determined by densitometry, and calibration curves were constructed by plotting pixel intensity against the amount of lipid standard applied. Lipid content in tissue samples was subsequently calculated from the corresponding calibration curves and normalized to tissue weight (supplementary Fig. 1).To visualize the lipid classes, the silica plates were immersed for 10 s in a carbonization solution composed of 8% CuSO₄ and 10% H₃PO₄ (v/v), then heated at 110 °C for 20 min [33]. Finally, the plates were analyzed by densitometry using the Image Master TotalLab software (TotalLab, Newcastle, England, UK).

### Statistical analysis

Statistical analyses were performed using Prism GraphPad (GraphPad Software, version 8.0, LLC, San Diego, California, USA). Data were initially tested for normal distribution using the Shapiro–Wilk test. As the data met normality assumptions, parametric tests were applied. None of the animals was excluded from the statistical analysis.

Sample size was determined using G*Power software (version 3.1-Universität Düsseldorf, Germany). The calculation was based on the percentage of time spent at the open arms of the elevated plus maze, as reported in a previous study conducted under the same experimental conditions [27, 30]. A two-tailed Student’s t-test was selected with an effect size (Cohen’s d) of 1.95, an α error probability of 0.05, and a desired power (1 − β) of 0.95. The analysis indicated a minimum sample size of eight animals per group, resulting in an achieved power of 0.952 [34].

Behavioral data were analyzed using unpaired Student’s t tests to compare control and TBI groups. For lipid analyses, multiple unpaired t-tests were performed to compare lipid classes between groups. When applicable, correction for multiple comparisons was applied using the Holm–Sidak method. Data are presented as the mean ± standard error of the mean (SEM). Statistical significance was set at *p* < 0.05.

Pearson’s correlation analysis was used to examine relationships between behavioral parameters and lipid measurements. Behavioral data obtained from the elevated plus maze were correlated with lipid classes quantified in the hippocampus, prefrontal cortex, and serum. Correlation coefficients (*r*), corresponding *p-values*, and sample size (*n*) were calculated. Statistical significance was defined as *p* < 0.05.

## Results

### Behavioral alterations following mild traumatic brain injury

**Fig. 2:**
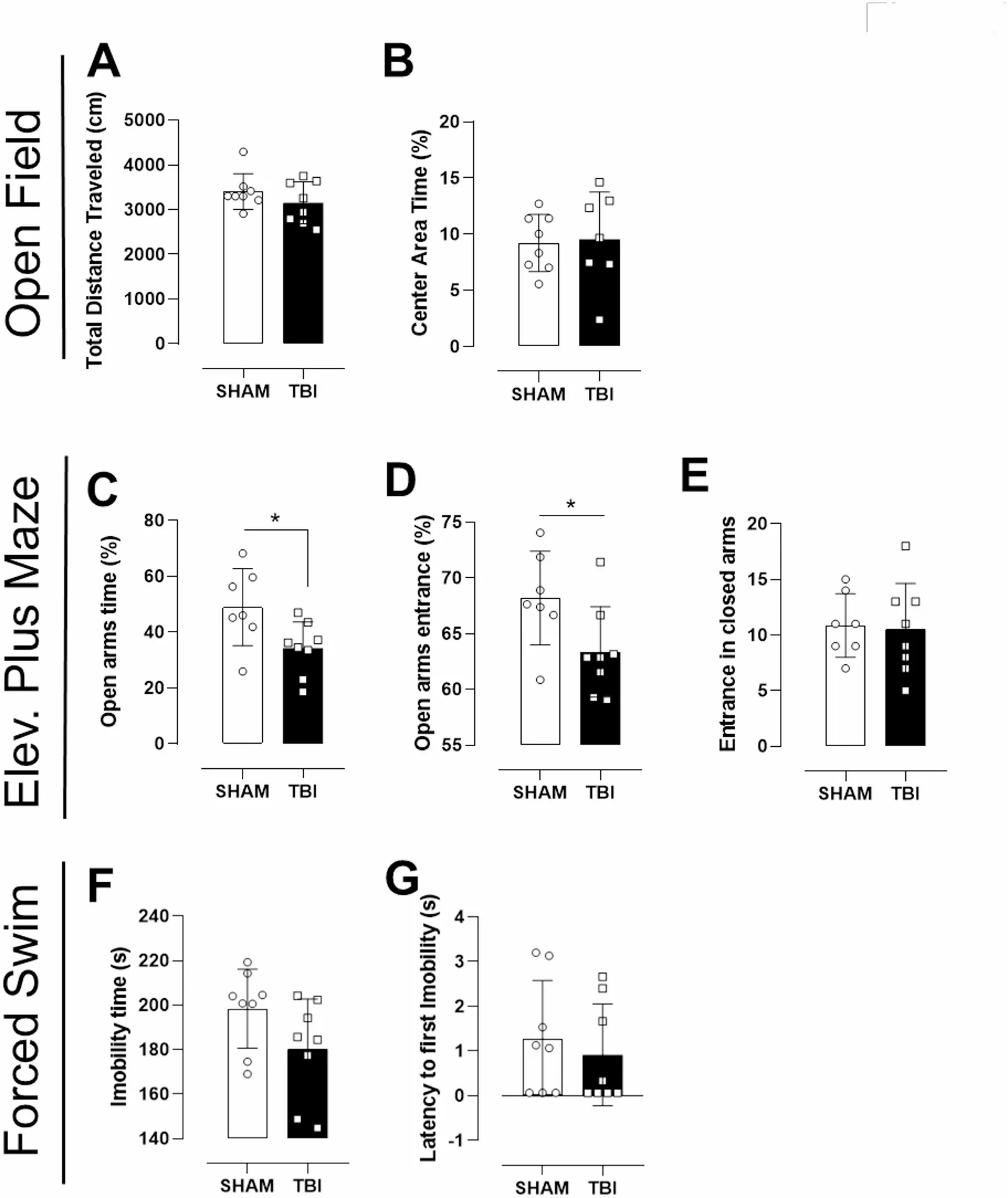
Behavioral analysis of male mice following mTBI. Male C57BL/6 wild-type mice (20–30 g), aged 10–12 weeks, were subjected to mild traumatic brain injury. Forty-eight hours after mTBI, mice were evaluated in the open field test, and 72 h post-injury, they were subjected to the elevated plus maze (EPM) and forced swim tests. Panels (**A**–**B**) show the parameters assessed in the open field test. Panels (**C**–**E**) show the parameters evaluated in the elevated plus maze, and panels (**F**–**G**) show the parameters measured in the forced swim test. Data are presented as mean ± standard error of the mean (SEM). Statistical analysis was performed using Student’s t-test, with significance set at *p* < 0.05

Regarding behavioral assessments, no significant differences were observed between TBI and sham animals in the open field test, indicating no major motor impairment (Fig. 2, a-b). Importantly, TBI mice displayed a significant decrease in the percentage of time spent (Fig. 2, c) and percentage of entries (Fig. 2, d) in the open arms of the elevated plus maze compared to sham, suggesting the development of anxiety-like behavior following the brain injury. No significant changes were observed in the absolute number of entries in the closed arms (Fig. 2, d), reinforcing the lack of impairment in locomotor activity. No differences were observed in the forced swimming test (Fig. 2, f-g).

### Mild traumatic brain injury promotes significant alterations in hippocampal lipid composition

**Fig. 3:**
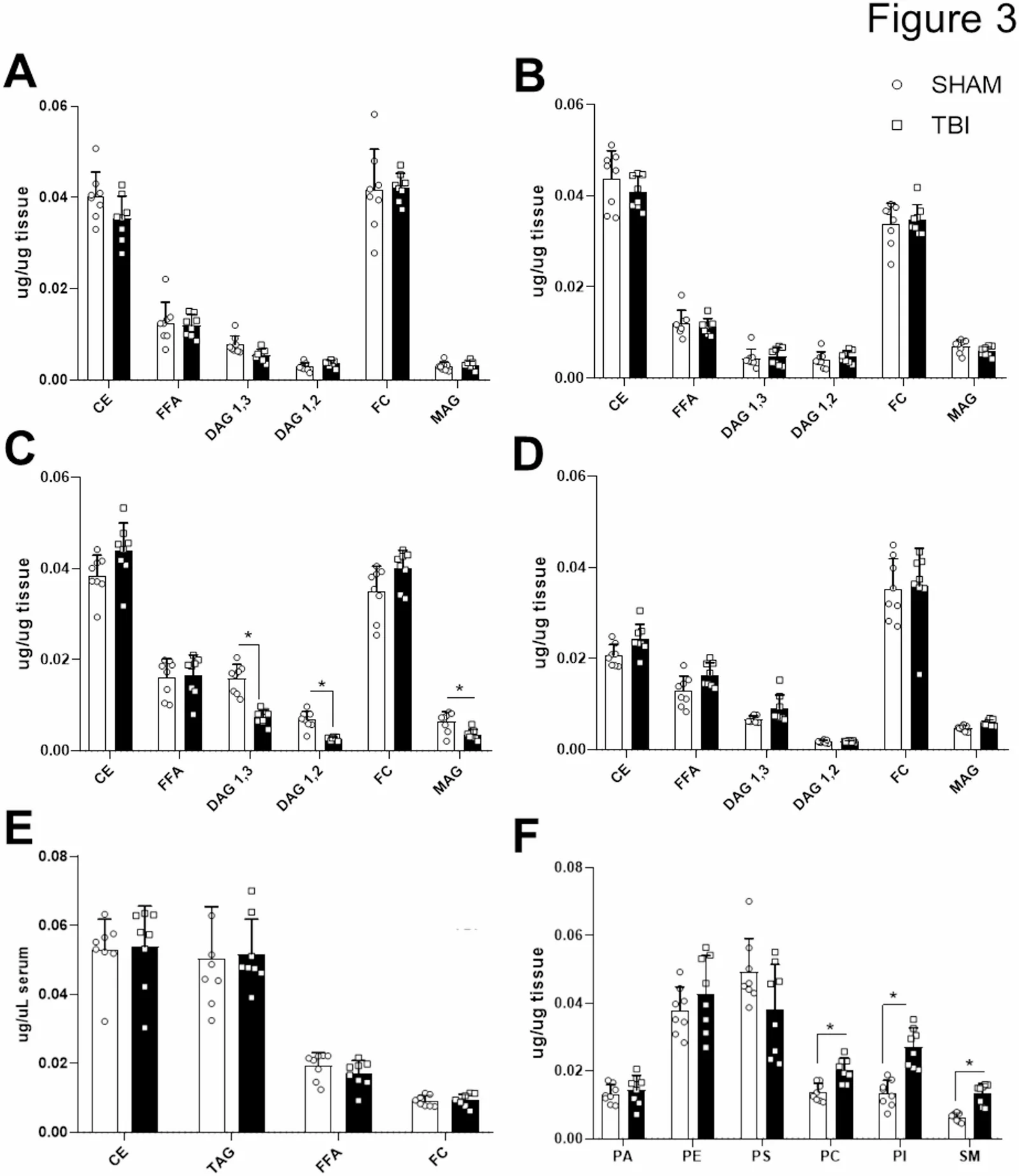
Lipid class analysis in the prefrontal cortex, hippocampus, and serum of male mice subjected to mTBI. At the end of the behavioral assessments (72 h after mTBI), animals were euthanized, and the prefrontal cortex, hippocampus, and serum were collected. Samples were subjected to lipid extraction and analyzed by thin-layer chromatography. Panels (**A**–**B**) show lipid classes in the ipsilateral and contralateral prefrontal cortex, respectively. Panels (**C**–**D**) show lipid classes in the ipsilateral and contralateral hippocampus, respectively. Panel (**E**) shows lipid classes separated in serum. Panel (**F**) shows the separation of phospholipids specifically in the ipsilateral hippocampus. Abbreviations: CE (Cholesterol Ester), FFA (Free Fatty Acids), DAG 1,3 (Diacylglycerol 1,3), DAG 1,2 (Diacylglycerol 1,2), FC (Free Cholesterol), MAG (Monoacylglycerol), PA (Phosphatidic Acid), PE (Phosphatidylethanolamine), PS (Phosphatidylserine), PC (Phosphatidylcholine), PI (Phosphatidylinositol), SM (Sphingomyelin). Data are presented as mean ± standard error of the mean (SEM). Statistical analysis was performed using multiple t-tests, with significance set at *p* < 0.05

**Fig. 4:**
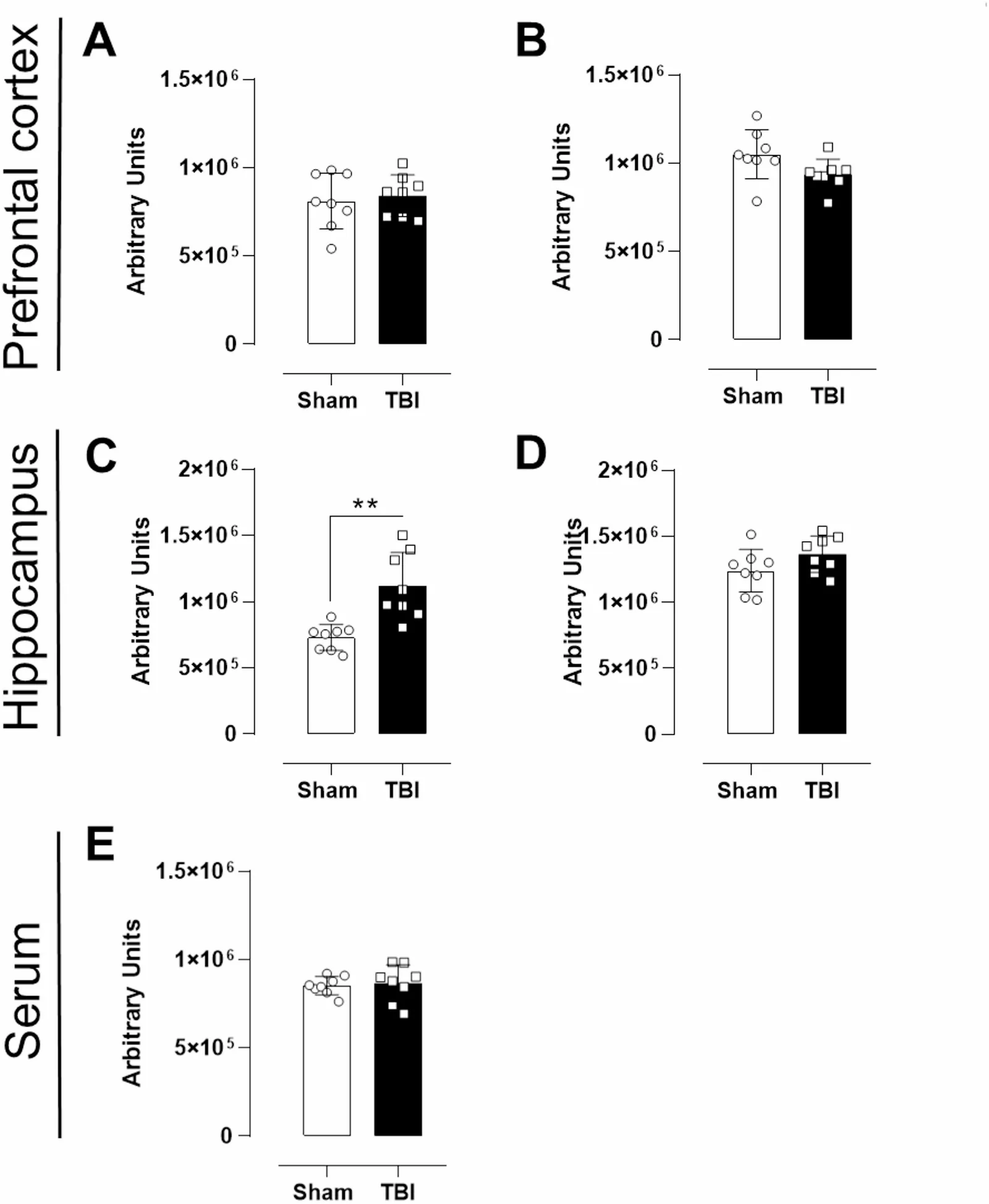
Total phospholipid analysis in the prefrontal cortex, hippocampus, and serum of male mice subjected to mTBI. At the end of the behavioral assessments, animals were euthanized, and the prefrontal cortex, hippocampus, and serum were collected. Samples were subjected to lipid extraction and analyzed by thin-layer chromatography. Panels (**A**–**B**) show total phospholipids in the ipsilateral and contralateral prefrontal cortex, respectively. Panels (**C**–**D**) show total phospholipids in the ipsilateral and contralateral hippocampus, respectively. Panel (**E**) shows total phospholipids in serum. Data are presented as mean ± standard error of the mean (SEM). Statistical analysis was performed using Student’s t-test, with significance set at *p* < 0.05

Separation of lipid classes by thin-layer chromatography revealed significant differences between sham and TBI mice. Reduced levels of neutral lipids, including diacylglycerol (DAG) (26.15 *±* 0.8 ng/µg tissue vs. 6,8 *±* 0.5 ng/µg tissue, *p* < 0.0001) and monoacylglycerol (MAG) (3.6 *±* 0.14 ng/µg tissue vs. 6.4 *±* 0.26 ng/µg tissue, *p* < 0.0001), were detected in the ipsilateral hippocampus of TBI mice compared to sham (Fig. 3, c). No significant differences were observed in lipid classes between the ipsilateral and contralateral prefrontal cortex (Fig. 3, a-b), nor in the contralateral hippocampus (Fig. 3, d). Regarding the total phospholipid content, it was significantly increased in the ipsilateral hippocampus of TBI mice relative to sham (1,120,698 *±* 100,354 au vs. 730,222 *±* 121,673 au, *p* = 0.0012, Fig. 4, c). However, no significant differences were observed in the total phospholipids in the ipsilateral and contralateral prefrontal cortex (Fig. 4, a-b), nor in the contralateral hippocampus (Fig. 4, d). When total phospholipids were further separated in the ipsilateral hippocampus, higher levels of phosphatidylcholine (20.4 *±* 1.5 ng/µg tissue vs. 13.75 *±* 3.4 ng/µg tissue, *p* = 0.000745), phosphatidylinositol (27.01 *±* 2.6 ng/µg tissue vs. 13.41 *±* 1.8 ng/µg tissue, *p* = 0.000066), and sphingomyelin (13.31 *±* 4.3 ng/µg tissue vs. 6.41 *±* 0.21 ng/µg tissue, *p* = 0.000033) were observed in TBI animals (Fig. 3, f).

### Associations between anxiety-like behavior and lipid classes across tissues

There were significant correlations between anxiety-like behavior and lipid classes across distinct tissues in the mTBI group. In the ipsilateral hippocampus, time spent in the open arms of the elevated plus maze was positively correlated with diacylglycerol 1,2 (*r* = 0.73, *p* = 0.039), monoacylglycerol (*r* = 0.91, *p* = 0.005), cholesterol esters (*r* = 0.87, *p* = 0.012, Table 1 )Analyses of the contralateral hippocampus revealed positive associations between time spent in the open arms with free cholesterol content (*r* = 0.76, *p* = 0.048, Table 1). At the cortical level, anxiety-related behavioral parameters correlated with cholesterol ester levels, including both open arm time (*r* = −0.83, *p* = 0.021) and the number of open arm entrances (*r* = −0.93, *p* = 0.002). Serum lipid analysis demonstrated positive correlations between open arm time and circulating free cholesterol (*r* = 0.84, *p* = 0.010) as well as free fatty acids (*r* = 0.75, *p* = 0.031). Finally, within the sham group, open arm entries were positively correlated with phospholipid levels in the contralateral hippocampus (*r* = 0.80, *p* = 0.031) and the ipsilateral hippocampus (*r* = 0.93, *p* = 0.003) (Table 1). A complete summary of correlation coefficients, p-values, and sample sizes is provided in Table 1. Scatter plots illustrating the distribution of individual data points and the corresponding linear regression analyses are presented in Supplementary Fig. S2.

**Table 1:** Correlations between anxiety-like behavioral parameters and lipid classes across brain regions and serum following mild traumatic brain injury

Behavioral parameter	Lipid class	Brain region/Serum	Pearson *r*	*p* value
TBI group				
Open arm time	Diacylglycerol 1,3	Hippocampus (ipsilateral)	0.7325	0.0388
Open arm time	Monoacylglycerol	Hippocampus (ipsilateral)	0.9071	0.0048
Open arm entrances	Cholesterol esters	Hippocampus (ipsilateral)	0.8663	0.0117
Open arm time	Free cholesterol	Hippocampus (contralateral)	0.7586	0.0481
Open arm time	Cholesterol esters	Cortex (contralateral)	−0.8293	0.0210
Open arm entrances	Cholesterol esters	Cortex (contralateral)	−0.9306	0.0024
Open arm time	Free cholesterol	Serum	0.8369	0.0096
Open arm time	Free fatty acids	Serum	0.7536	0.0308
Sham group				
Open arm entrances	Phospholipids	Hippocampus (contralateral)	0.7995	0.0309
Open arm entrances	Phospholipids	Hippocampus (ipsilateral)	0.9273	0.0026

Pearson’s correlation coefficients (r) and corresponding p values are shown for significant associations identified within the mTBI and sham groups. Behavioral parameters obtained from the elevated plus maze were correlated with lipid classes quantified in the hippocampus (ipsilateral and contralateral), cortex (contralateral), and serum. Statistical significance was set at *p* < 0.05, *n* = 8/group.

## Discussion

The current study demonstrated that mTBI induces region-specific alterations in brain lipid classes within the acute phase (72 h) that correlate with anxiety-like behavioral changes. The most prominent biochemical alterations were observed in the ipsilateral hippocampus, a region consistently reported to be highly vulnerable to mechanical and metabolic insults following experimental TBI [35, 36]. Previous studies from our group and others have shown that intense secondary injury responses, including neuroinflammation, oxidative stress, and metabolic disruption, characterize the early post-injury phase [37–39]. Our findings expand this framework by showing that lipid profile alterations are already present at this early stage, reinforcing the importance of investigating metabolic processes associated with and at the peak of secondary injury cascades.

Consistent with previous findings from the same weight-drop mTBI model [27], injured animals exhibited reduced exploration of the open arms in the elevated plus maze, indicative of an anxiety-like phenotype. No significant alterations in spontaneous locomotor activity were detected, as evidenced by the absence of differences in open field performance and in the number of closed-arm entries in the elevated plus maze. These observations indicate preserved locomotor function and support the interpretation that the behavioral alterations observed following mTBI are attributable to anxiety-like behavior rather than impaired motor performance. Analysis of lipid classes revealed region-dependent alterations following mTBI, with more pronounced changes detected in the hippocampus. We observed a reduction in neutral lipid species, including DAG and MAG, accompanied by an increase in total phospholipids and specific phospholipid subclasses. Alterations in hippocampal lipid composition following TBI have been reported in previous studies, but these changes are often associated with long-lasting metabolic and structural changes in injured brain tissue [13, 20]. Our findings extend these observations by demonstrating that altered lipid composition is already evident during the acute phase, supporting the notion that such changes emerge early and may persist.

From a metabolic standpoint, the reduction of DAG and MAG levels observed in the hippocampus suggests disturbances in key lipid intermediates following mTBI. These lipid species are involved in membrane dynamics and cellular signaling pathways [40, 41]. Alterations in their levels may reflect adaptive or maladaptive biochemical responses to injury. Although the present study did not assess fatty acid composition of DAG and MAG, the observed results indicate that lipid metabolic pathways are affected following mild TBI. In parallel, the increase in phospholipid species, such as phosphatidylinositol and sphingomyelin, is consistent with membrane remodeling processes observed in neurological conditions, including TBI [15, 20, 24, 42, 43].

TBI induces early lipid remodeling within 1–3 days post-injury, including cell–type–specific phospholipid alterations in the brain [23, 24], increased PUFA-containing DAG in serum (3 and 7 days post-injury) [14], and elevated ether phospholipids in plasma (1, 3, 7, 14, and 28 days post-injury) [22], supporting the notion that acute TBI is accompanied by dynamic and regionally diverse lipid changes. These previous investigations have largely focused on describing biochemical changes, identifying potential biomarkers, or elucidating mechanisms of tissue damage and repair. Comparatively little attention has been given to the relationship between lipid disturbances and behavioral manifestations after injury. In this context, the present study extends the existing literature by examining not only regional lipid alterations following mTBI but also their potential association with anxiety-like behavioral outcomes.

To a lesser extent, lipid alterations were also detected in the prefrontal cortex, indicating that the effects of mTBI are not restricted to the site of impact or to a single brain region. The involvement of cortical areas has been described in other experimental models of mTBI and has been linked to alterations in behavioral and cognitive responses [8, 44–46]. Accordingly, correlation analysis revealed significant associations between anxiety-like behavioral parameters and specific lipid classes in the hippocampus, prefrontal cortex, and serum (Table 1). Evidence from experimental and clinical studies indicates that lipid alterations are associated with anxiety-related phenotypes in neuropsychiatric conditions, including stress-related disorders [47, 48]. Different studies have also reported associations between alterations in phospholipids, fatty acid metabolism, endocannabinoid signaling, and the development of anxiety-like phenotypes [15, 19, 20, 42, 47–49]. These observations support the notion that lipid homeostasis may influence behavioral outcomes. Whether similar relationships exist following TBI remains uncharacterized, particularly during the acute post-injury period. An interesting observation from the correlation analyses was the positive association between hippocampal phospholipid levels and open-arm exploration in sham animals. In contrast, no such association was observed following mTBI, despite the increase in phospholipid levels detected in the ipsilateral hippocampus. Together, these findings suggest that trauma-induced phospholipid alterations may reflect injury-related metabolic responses distinct from the physiological relationships observed under basal conditions. While correlation analyses do not permit causal inference, these associations suggest that changes in lipid metabolism co-occur with behavioral alterations following mTBI and may reflect shared or interconnected pathological processes.

Limitations of the present study must be acknowledged. Behavioral assessments were performed without baseline measurements or the inclusion of a naïve control group. Although sham-operated animals are widely accepted as controls in closed-head mTBI models, the absence of pre-injury evaluations limits the ability to fully dissociate trauma-related effects from potential influences associated with experimental procedures and handling. This consideration is particularly relevant when interpreting subtle or negative behavioral findings, such as those obtained in the forced swim test.

Taken together, our findings indicate that mTBI is followed by anxiety-like behavioral alterations and region-specific changes in brain lipid composition during the acute post-injury phase, particularly in the hippocampus. These results add to a growing body of evidence indicating that lipid metabolism is altered following TBI and may contribute to behavioral outcomes. While further studies are required to define the precise molecular mechanisms involved, the present data support the relevance of lipid metabolic alterations as an important component of the cerebral response to mild traumatic injury.

## Supplementary Information

Below is the link to the electronic supplementary material.

Supplementary Material 1

Supplementary Material 2

## Data Availability

No datasets were generated or analysed during the current study.
